# Multi-decadal tree-ring stable isotope records of apple and pear trees indicate coherent ecophysiological responses to environmental changes in alpine valleys

**DOI:** 10.3389/fpls.2024.1471415

**Published:** 2025-01-10

**Authors:** Nilendu Singh, Massimo Tagliavini, Enrico Tomelleri, Leonardo Montagnani

**Affiliations:** ^1^ Faculty of Agricultural, Environmental and Food Sciences, Free University of Bolzano, Bolzano, Italy; ^2^ Wadia Institute of Himalayan Geology, Dehradun, India

**Keywords:** dendrochronology, ecophysiology, WUE, climate-carbon response, Italian Alps

## Abstract

The ecophysiological and ecohydrological impacts of climate change and progressively increasing atmospheric carbon dioxide (CO_2_) concentration on agroecosystems are not well understood compared to the forest ecosystems. In this study, we utilized the presence of old apple and pear trees in the alpine valleys of Northern Italy (maintained for cultural heritage purposes) to investigate climate-scale physiological responses. We developed long-term tree-ring stable isotopic records (δ^13^C and δ^18^O) from apple (1976-2021) and pear trees (1943-2021). This allowed the reconstruction of key ecophysiological processes like the variations in intrinsic water use efficiency (*i*WUE), and we investigated how these trees responded to climate and CO_2_ changes over decades. Results showed a slight declining trend in carbon discrimination (*Δ* ^13^C) while intercellular CO_2_ concentration (*C*i) for both species has been increasing since the late 1980s. Concurrently both species exhibited a rising trend in *i*WUE, with apple trees demonstrating higher efficiency, which appears to be primarily driven by the CO_2_-fertilization effect. The concomitant trends in tree-ring δ^18^O suggested a relatively stable local hydroclimate during the study period with some species-specific responses. Analyses further revealed that minimum growing season temperature, not precipitation was the most significant factor influencing the rise in *i*WUE alongside with CO_2_ fertilization effect. Analyses of species’ δ^13^C coupled with their respective δ^18^O confirmed that the rise in *i*WUE was due to increased carbon assimilation rather than a decline in evapotranspiration. Moreover, coupled δ^13^C–δ^18^O analyses suggested increasing trends in carbon assimilation, with apple trees showing higher inter-decadal variations. These long-term records provide a unique opportunity to test and calibrate how these systems respond to recent and anticipated climate change.

## 1 Introduction

The forest and agroecosystems in the European Alps play a very important role in providing food, goods, and ecosystem services (Stenger et al., 2009). However, concurrent climatic change and continually increasing atmospheric CO_2_ concentration are expected to strongly affect the ecophysiology of these ecosystems, which could alter their productivity. The fertilizing effects of rising CO_2_ levels, along with nitrogen depositions and increasing temperatures, have been shown to positively affect the productivity of European forests (Hyvönen et al., 2007; Giammarchi et al., 2017). Important studies that analyzed stable tree-ring isotopes across European forests have revealed valuable insights into forest ecosystem functioning and responses to climate change, including the CO_2_-fertilization effect (Huang et al., 2024). The intrinsic water use efficiency (*i*WUE: ratio of photosynthesis to stomatal conductance) along with forest transpiration was found to increase over the 20^th^ century with a consistent south-to-north gradient, which largely depended upon local growth limiting factors (Peñuelas et al., 2011). Across Europe, the strongest increase in *i*WUE was observed in the water-limited temperate forests in the central region (Saurer et al., 2014). Concerning the magnitude, mechanisms, and spatial patterns, the impact of climate change on European forests is quite diverse depending upon the geography and local growth limiting factors (Saurer et al., 2014; Frank et al., 2015).

Nevertheless, the response of tree physiology to increasing CO_2_ levels is far from being a straightforward one, indeed it strongly depends on local conditions with a species-specific response (Huang et al., 2024). It could interact with other climate drivers, such as warming-induced soil drying and physiological acclimation to high CO_2_ levels (Saurer et al., 2014; Frank et al., 2015). Consequently, a global analysis of tree-ring isotope datasets indicates diminishing CO_2_-driven gains in *i*WUE, in which deciduous species contributed more than conifers to the recent slowdown (Adams et al., 2020). Particularly, European forests at the northern periphery show a progressively diminishing response to increasing CO_2_ concentration (Waterhouse et al., 2004). Moreover, the debate on the relative roles of enhanced photosynthesis vs reduced stomatal conductance in the global trends of *i*WUE has been tried to settle by combining tree‐ring δ^13^C and δ^18^O datasets with a water–carbon optimality model (Lin et al., 2022; Walker et al., 2021).

Conversely, the response of climate change including CO_2_-fertilization effect on the physiological functioning of economically important agroecosystems is less explored. This is primarily because of the lack of old wild or cultivated trees having long-term tree-ring width or isotopic records. Tree-ring stable isotopic records (δ^13^C and δ^18^O) differ from classical dendrochronological variables (such as width) as they reflect more directly the plant’s physiological response to climate and environmental variables (Wang et al., 2019). δ^13^C values depend on factors affecting the photosynthetic uptake of CO_2_ and are mainly controlled by stomatal conductance and the rate of carboxylation during photosynthesis. Whereas, δ^18^O values are constrained by the isotopic ratio of the source water and locally integrate the stomatal response to vapor pressure deficit via leaf water enrichment, coupled with transpiration. These factors controlling isotopic fractionation are closely related to the meteorological variables (McCarroll and Loader, 2004; Battipaglia et al., 2013; Gagen et al., 2022). In this context, tree isotopes provide precise, reliable, large-scale, and long-term information to advance our understanding of ecosystem functioning, carbon – water cycling and to reconstruct key metrics and processes for the decades preceding observational data (Babst et al., 2014). Moreover, dual isotope analysis (δ^18^O – δ^13^C) provides a physiological basis to understand carbon – water processes including stomatal conductance and the effect of climate warming and CO_2_ –fertilization (Siegwolf et al., 2023). The physiological interpretation of tree-ring δ^18^O is indeed complex (Lin et al., 2022). Yet, it remains the only proxy in conjunction with δ^13^C, which could be used to reconstruct mechanisms through which *i*WUE changes in response to climatic drivers, including atmospheric CO_2_ (Siegwolf et al., 2023).

**Figure 1:**
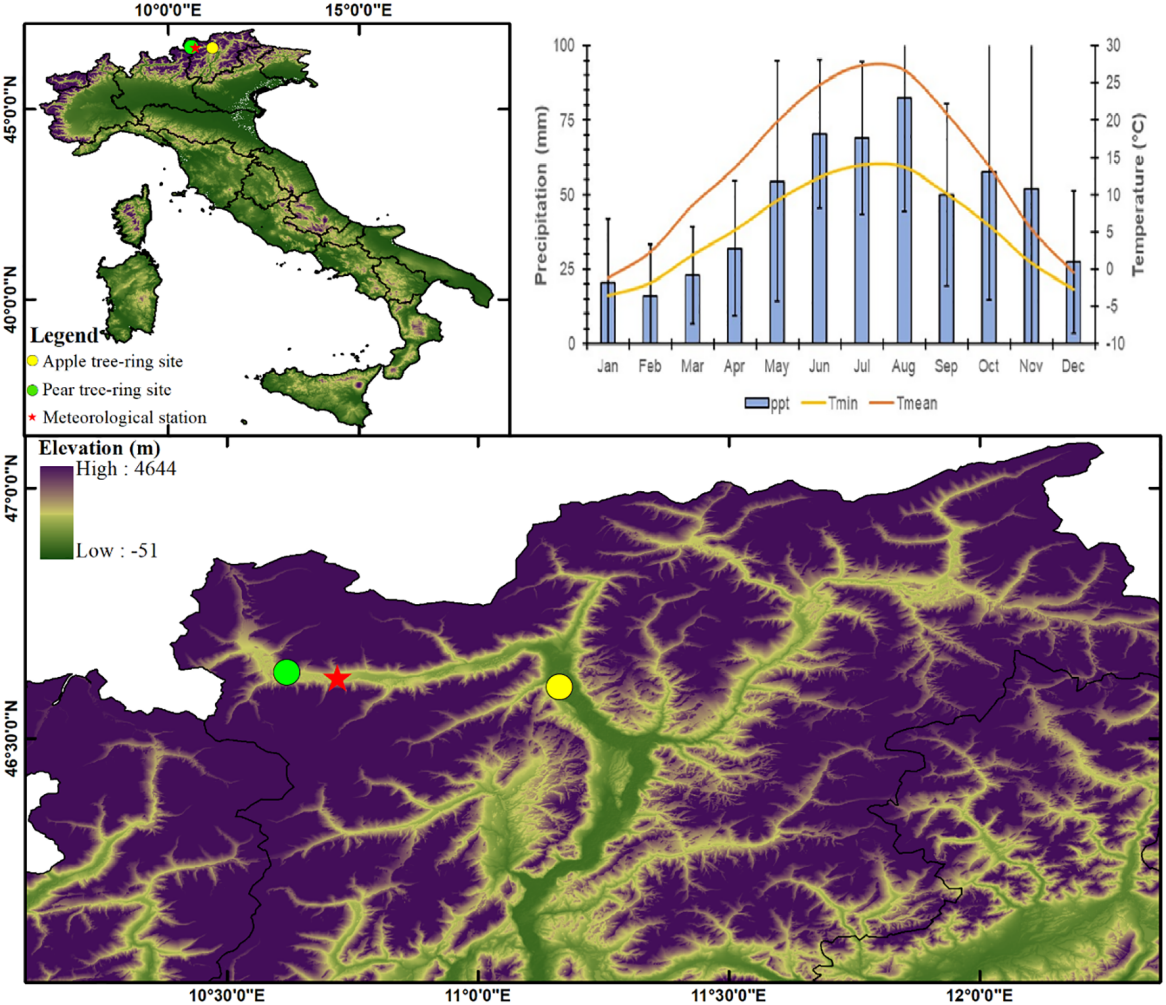
Location map of studied apple and pear orchards in the alpine valleys of the Italian Alps from where tree cores have been collected. The meteorological plot indicates regional climatology (Meteorological station: Silandro; Precipitation: 1981-2021; Temperature: 1988-2021).

In this study, we generated long-term stable isotope (δ^13^C and δ^18^O) records of unique and old apple (1976-2021) and pear trees (1943-2021), which constitute major agro-economic crops in the Italian Alps (Alto Adige, Northern Italy, **Figure 1**). The alpine valleys of the region is a major apple production center in the country, where we analyzed δ^13^C and δ^18^O chronologies to understand how these trees responded to climate and CO_2_ changes over decades. This study specifically aims to reconstruct δ^13^C-based ecophysiological processes and carbon-water coupling process (*i*WUE) and to investigate long-term climate – carbon responses. This approach lays the foundation for using wood carbon-oxygen stable isotope analysis to understand the meteorological constraints on fruit tree production potential.

## 2 Materials and methods

### 2.1 Study sites and climate

The study sites are located in valleys of the eastern Alps in South Tyrol, Italy (**Figure 1**). This region is a major apple producer, boasting around 18,000 hectares of apple orchards that contribute roughly half of Italy’s apple production. In the past, pear trees were also widely cultivated, but this practice declined significantly by the 1970s. Land previously used for pears now primarily houses apple orchards. Current apple yields in the region average around 55 tons per hectare. Due to the relatively short lifespan of apple and pear orchards (around 20-30 years), they are not ideal for studying long-term climate impacts. To address this challenge, we collaborated with experts from South Tyrolean Fruit Tree Cultivation Museum to access two rather unique sites featuring old veteran apple and pear trees maintained for cultural heritage purposes. The apple orchard, within the municipality of Lana (46.60° N, 11.16° E, 310 m asl), featuring trees of the variety “*Gravenstein*” grafted on seedling rootstocks, was established around 1976. The pear orchard is located in the municipality of Prato allo Stelvio (46.63° N, 10.61° E, 884 m asl). Trees of the variety “*Bartlett*”, grafted on seedling rootstocks, were planted between 1928 and 1938. The pear site sampling also included a monumental tree of more than 200 years old belonging to the variety “*Pala Birne*”. Both orchards have received regular management practices since their establishment, including pruning, fertilization, irrigation, pest and disease control, and fruit harvesting. The soil in the sampling area has a sandy loam texture and is relatively fertile due to its high organic matter content.

Meteorological data from the Schlanders-Silandro station (46.62° N, 10.72° E, 698 m asl), located between the two sampling sites (**Figure 1**), suggests that the sites are energy-limited ecosystems, where temperature remains optimal only during the short growing season (April – September). These records indicate annual precipitation (1981 – 2021) in the range of 400 – 800 mm, of which, the growing season months (April – September) receive about 60% and the rest mostly as snowfall during winter. The mean annual temperature (1988 – 2021) varies between -1.2 and 27.3°C, which remains above 13°C during the growing season (**Figure 1**). The range of variation of minimum temperature is -3.5 to 14.0°C, which remains above 5°C during the growing season (**Figure 1**). In recent decades, temperature trends have generally been upward, with an overall warming trend observed across the region. The average temperature in Europe has increased by around 1.5°C since the pre-industrial era, and the warming trend has been more pronounced in winter than in summer. According to the European Environment Agency, overall precipitation has remained relatively stable, with an increase of around 5% since the pre-industrial era.

### 2.2 Tree-ring stable isotope chronologies and computations

For each species, five trees were randomly selected and from each tree, two cores were extracted at 0.5 m from the ground using a 10 mm diameter increment borer. These increment cores were air-dried and glued on wooden supports. The cross-sectional surface of the cores was sanded by increasingly fine sandpapers until growth rings were visible and finally digitalized with a high-resolution scanner (2400 d.p.i.; Epson Expression 10000XL, Long Beach). A standard image was created for each sample and all images were saved into a graphic file format for further analysis. Subsequently, the determination of the tree ring width of each sample was performed with the Coo-recorder software (Cybis Elektronik & Data AB, Saltsjöbaden, Sweden) at a precision level of 0.01 mm (García-Hidalgo et al., 2022).

We selected three individuals from each species based on the absence of biotic damages and on the chronological length for the isotopic analyses. Each year’s growth-ring was cut using a sharp razor blade under the binocular microscope and pooling was performed for the individual tree rings of corresponding age. We utilized the whole-wood for the stable isotope analyses (δ^13^C and δ^18^O) (Schollaen et al., 2017; Wieser et al., 2016). Stable isotope analyses were carried out at the Università degli studi della Campania “L. Vanvitelli” Dipartimento di Scienze e Tecnologie Ambientali Biologiche e Farmaceutiche, Caserta, Italy. The analytical precision was equal to or better than 0.2‰ for both the isotopes. The isotope ratios are presented in common δ-notation against international standard PDB and VSMOW respectively as:


(1) $$\delta^{13}\text{C}=\;\left[\frac{\left(\frac{13_{\text{C}}}{12_{\text{C}}}\right)\text{sample}}{\left(\frac{13_{\text{C}}}{12_{\text{C}}}\right)\text{PDB}}-1\right]\times 1000\;\left(‰\right)$$

(2) $$\delta^{18}\text{O}=\;\left[\frac{\left(\frac{18_{\text{O}}}{16_{\text{O}}}\right)\text{sample}}{\left(\frac{18_{\text{O}}}{16_{\text{O}}}\right)\text{VSMOW}}-1\right]\times 1000\;\left(‰\right)$$

Where, (^13^C/^12^C) sample and (^13^C/^12^C) PDB are heavy to light carbon isotope ratios in the wood sample and the standard (Vienna Pee Dee Belemnite), respectively. (^18^O/^16^O) sample and (^18^O/^16^O) VSMOW are heavy to light oxygen isotope ratios in the wood sample and the international standard (Vienna Standard Mean Ocean water) respectively.

Discrimination against ^13^C (Δ^13^C, ‰*)* during carbon fixation by trees was computed by using atmospheric (δ^13^C_atm_) and tree-ring (δ^13^C_plant_) δ^13^C as:


(3) $$\Delta^{13}\text{C}=\;\frac{\text{δ}13_{\text{Catm}}-\text{δ}13_{\text{Cplant}}}{1+\text{δ}13_{\text{Cplant}}}$$

Where, (δ^13^C_atm_) and (δ^13^C_plant_) are fractional differences in isotopic composition (^13^C/^12^C) in atmospheric CO_2_ and that of tree-ring wood. We compiled δ^13^C_atm_ from McCarroll and Loader (2004) up to the year 2004, and after that was derived from Belmecheri and Lavergne (2020) (https://scrippsco2.ucsd.edu/data/). A widely accepted procedure to correct tree-ring isotope chronology for the incorporation of isotopically light carbon released by the burning of fossil fuels and increasing CO_2_ concentration was adopted (McCarroll and Loader, 2004). The correction procedure has the advantage of being an objective one as it effectively removes any declining trend in the δ^13^C series post AD 1850, which is attributed to physiological response to increased atmospheric CO_2_ concentrations (McCarroll and Loader, 2004).

Carbon isotopic discrimination (Δ^13^C) is related to intercellular CO_2_ (C*i*) and atmospheric CO_2_ (C*a*) concentration as:


(4) $$\Delta^{13}C=a+\left(b-a\right)\left(\frac{Ci}{Ca}\right)$$

Where, ‘*a*’ is the fractionation factor during intercellular diffusion (−4.4‰), and ‘*b*’ is the fractionation factor during carboxylation (−27‰) (Farquhar et al., 1982). The ratio of C*i* and C*a* was determined as:


(5) $$\frac{Ci}{Ca}=\frac{\delta 13C_{plant}-\delta 13C_{atm}+a}{a-b}$$

or,


$$\frac{Ci}{Ca}=\frac{\Delta^{13}C-a}{b-a}$$

Using C*i* and C*a* values, intrinsic water use efficiency (*i*WUE) was calculated as:


(6) $$iWUE=\frac{Ca-Ci}{1.6}$$

To substantiate our inferences, we computed a standardized carbon-to-oxygen isotope difference index for all species over the entire observation period, following the model of Scheidegger et al. (2000):


(7) $$\left(C-O\;Difference\;Index\right)n=\left(\delta^{13}C_{zscore}\right)n-\;\left(\delta^{18}O_{zscore}\right)n$$

Here, *n* is the year. Both corrected δ^13^C and δ^18^O chronologies were transformed into z-scores (based on the long-term mean and std. dev.) and analyzed in pairs for each crop. This index allows tracking year-by-year changes in trees’ physiological conditions induced by changes in stomatal conductance and photosynthetic capacity. The index assumes values close to 0 when both isotope ratios show similar values, indicating either enhanced stomatal conductance (both isotopic values are negative) or reduced stomatal conductance (both isotopic values are positive). Positive index values indicate high photosynthetic capacity (high δ^13^C and low δ^18^O), while negative values indicate low photosynthetic capacity (low δ^13^C and high δ^18^O) (Scheidegger et al., 2000; Siegwolf et al., 2023).

### 2.3 Statistical analyses

To understand relationships between δ^13^C-based processes (C*i*, Δ^13^C, *i*WUE*)* in the two species and the regional climate (Precipitation and Temperature: Mean, Max., Min.), simple Pearson correlations were applied with a response-function approach. The confidence intervals of correlations were analyzed at 95% and 99% levels. This helped to investigate the correlations with monthly climatic averages in the species. To corroborate and stabilize these relations further, we plotted 3-month moving correlation coefficients between physiological processes and monthly hydroclimatic data (precipitation: 1982-2021; temperature: 1988-2021). The response function analysis for the species was plotted from October of the previous growth year to September of the current year (pOct-Sep). To test the relative importance of climate parameters including atmospheric CO_2_ levels, multiple linear regression models with *i*WUE as the response variables, and temperature (mean, max, min), precipitation, and atmospheric CO_2_ as continuous predictor variables, were built.

To test the significance of the slopes (*p*-values), we first computed Mean Square Error (MSE) as: $MSE=\left(1-R^{2}\right)\times Var\;\left(y\right)$ .

Then, the Standard Error (SE) of the slope was computed as: $\sqrt{\frac{MSE}{\left(n-1\right)\times Var\left(x\right)}}$ ; and the t-statistic was calculated as: $t=\frac{slope}{SE}$ .

Finally, using the t-distribution, we calculated the two-tailed *p*-value for the respective t-statistics and degrees of freedom (*df* = n – 2).

We used ‘lm’ function from the R statistical computing environment (R development Core team, 2015). The relative importance of significant terms was obtained by applying function ‘calc.relimp’ using default options (**Supplementary Table S1**). The process reconstructions were standardized using Z-scores and smoothed with a 3-year running mean to assess common signals.

## 3 Results and discussion

### 3.1 Carbon isotope chronologies and climate response

**Figure 2:**
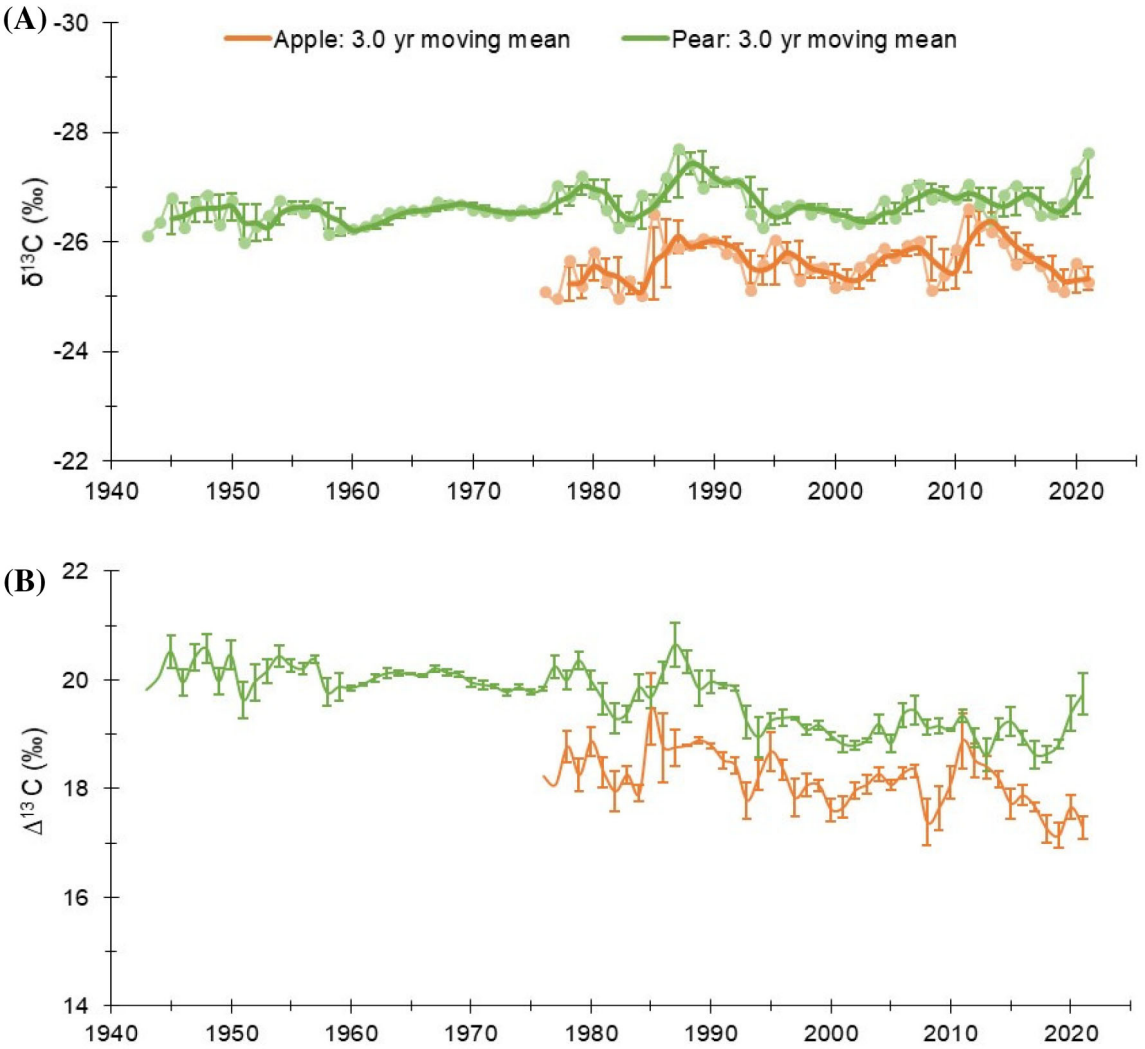
**(A)** δ^13^C chronologies of pear and apple trees (Pear: 1943 – 2021; Apple: 1976 – 2021). Faded line with dots denote annual values. Colored dark lines represent three year moving mean with three-year moving std. dev. **(B)** Annual atmosphere-to-plant ^13^CO_2_ discrimination (Δ^13^C) trends in the species with 3-year moving std. dev.

Over the study period, raw δ^13^C in tree-rings of pear (1943-2021) and apple (1976-2021) species exhibited a slight decreasing trend (**Figure 2**). The break-point analysis identifies the year of change in carbon isotopic composition in the species as the year 1987-88. Therefore, 1990 is assigned as a reference year for the analyses. For the common period, inter-species correlation was moderate at the inter-annual scale (*r* = 0.398, *p*< 0.001), which indicates the predominance of both species-specific and site-specific local effects on the assimilation process. The mean δ^13^C value of apple trees was −25.6‰ (std. dev.: 0.40‰), while for the pear trees, it was ∼1.0‰ lower (−26.6 ± 0.32‰) (**Figure 2A**; **Table 1**). This difference suggests a higher level of assimilation rate (isotope discrimination) in pear trees (**Figure 2B**). However, pear and apple trees have reportedly similar daily radiation use efficiency (Auzmendi et al., 2013). The long-term mean of δ^13^C (corrected for atmospheric CO_2_ increase) in apple trees was −23.5 ± 0.6‰, which increased slightly (−23.2 ± 0.52‰) after 1990. The long-term mean for the pear trees (−25.1 ± 0.72‰) increased by ~ 1‰ after 1990 (−24.3 ± 0.42‰) (**Supplementary Figure S1**). Considering the changes in atmosphere-to-plant ^13^CO_2_ discrimination (Δ^13^C), we found a higher (~2.0‰) level of discrimination in pear trees, which showed a similar temporal pattern to that of apple trees (**Figure 2**; **Table 1**). The corrections of the carbon isotope series of the two species for the physiological responses to increasing concentrations of atmospheric CO_2_ are illustrated in the supplementary figure (**Supplementary Figure S1**).

**Figure 3:**
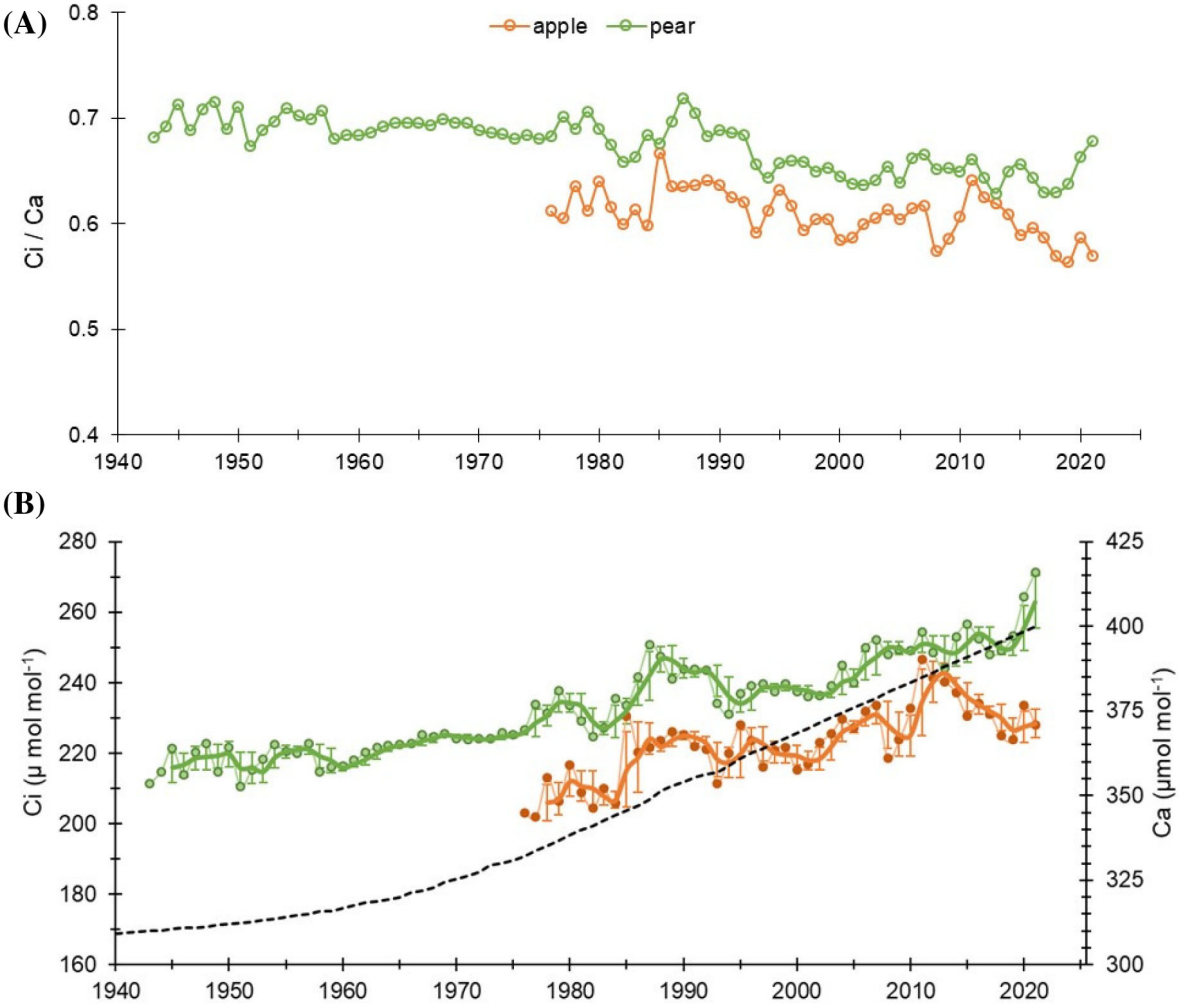
**(A)** Trends of C*i*/C*a* ratios in apple and pear trees during the study period. **(B)** Long-term C*i* trends in the species with respect to increasing atmospheric CO_2_ (C*a*: dashed black line). Light-colored lines with dots indicate annual values, while dark lines indicate 3-year moving average with 3-year moving std. dev.

A rising trend in intercellular CO_2_ (C*i*) in the two species was noted during the observation period. The mean C*i* value of the apple tree was 223 ± 10.3 µmol mol^-1^, while for the pear trees, it was 233 ± 13.6 µmol mol^-1^ (**Table 1**). Prior to 1990, the mean C*i* of apple trees was 207 µmol mol^-1^ (range: 181 – 230 µmol mol^-1^), which increased to 227 µmol mol^-1^ (211 – 247 µmol mol^-1^). While, for the pear trees, the mean C*i* before and after 1990 was 225 µmol mol^-1^ (211 – 251 µmol mol^-1^) and 245 µmol mol^-1^ (231 – 264 µmol mol^-1^), respectively (**Figure 3**). The trends in intercellular CO_2_ (C*i*) to atmospheric CO_2_ (C*a*), i.e., C*i*/C*a* ratio in both species were analogous to the trends in Δ^13^C. Nonetheless, a declining trend in both species is noticeable after 1990. Prior to 1990, the ratio for the apple trees was 0.62 (0.59 – 0.66), which decreased to 0.60 (0.56 – 0.64). While, in the pear trees the ratio before and after 1990 was 0.69 (0.66 – 0.71) and 0.65 (0.63 – 0.69), respectively. Fitted regression slopes for the C*i*/C*a* ratio (*p*< 0.05) in apple and pear trees prior to 1990 were 0.0021 (R^2^ = 0.2) and -0.0003 (R^2^ = 0.087), respectively. After 1990, magnitude of the slopes becomes more negative (Apple: -0.0011, R^2^ = 0.25; Pear: -0.0009, R^2^ = 0.28). Out of three theoretical scenarios of C*i*/C*a* ratios to CO_2_ rise (Panthi et al., 2020), a positive slope in apple trees suggests a ‘C*a* – C*i* = constant’ scenario prior to 1990 that changed to ‘C*i* = constant’ scenario after 1990. Conversely, the response of pear trees appears to be a ‘C*i* = constant’ scenario throughout the observation period. These results probably suggest a varying physiological response of tree species to atmospheric CO_2_ rise (**Figure 3**).

The Δ^13^C discrimination at the plant level is controlled by C*i*/C*a* ratio. This ratio could decline either because of low stomatal/mesophyll conductance to CO_2_ associated with water stress or low temperatures, or due to a high assimilation rate (McCarroll and Loader, 2004). At our sites, in the alpine valleys with frequent irrigation, water cannot be assumed to be a limiting factor. Growth at higher latitudes is generally limited by suboptimal temperatures for xylogenesis (i.e., formation of water conductive tissue), which remains almost optimal during the growing season. In energy-limited ecosystems at higher latitudes, climate warming may improve tree-water status where xylogenesis is temperature-limited and given that sufficient water is available. Therefore, increasing assimilation rates due to rising CO_2_ levels could be another reason for the observed declining C*i*/C*a* ratio in recent decades. The effect of CO_2_ fertilization has been observed globally, which is quite pervasive in European forests, particularly over the northern ecosystems (Mathias and Thomas, 2021; Waterhouse et al., 2004; Saurer et al., 2014; Frank et al., 2015).

**Figure 4:**
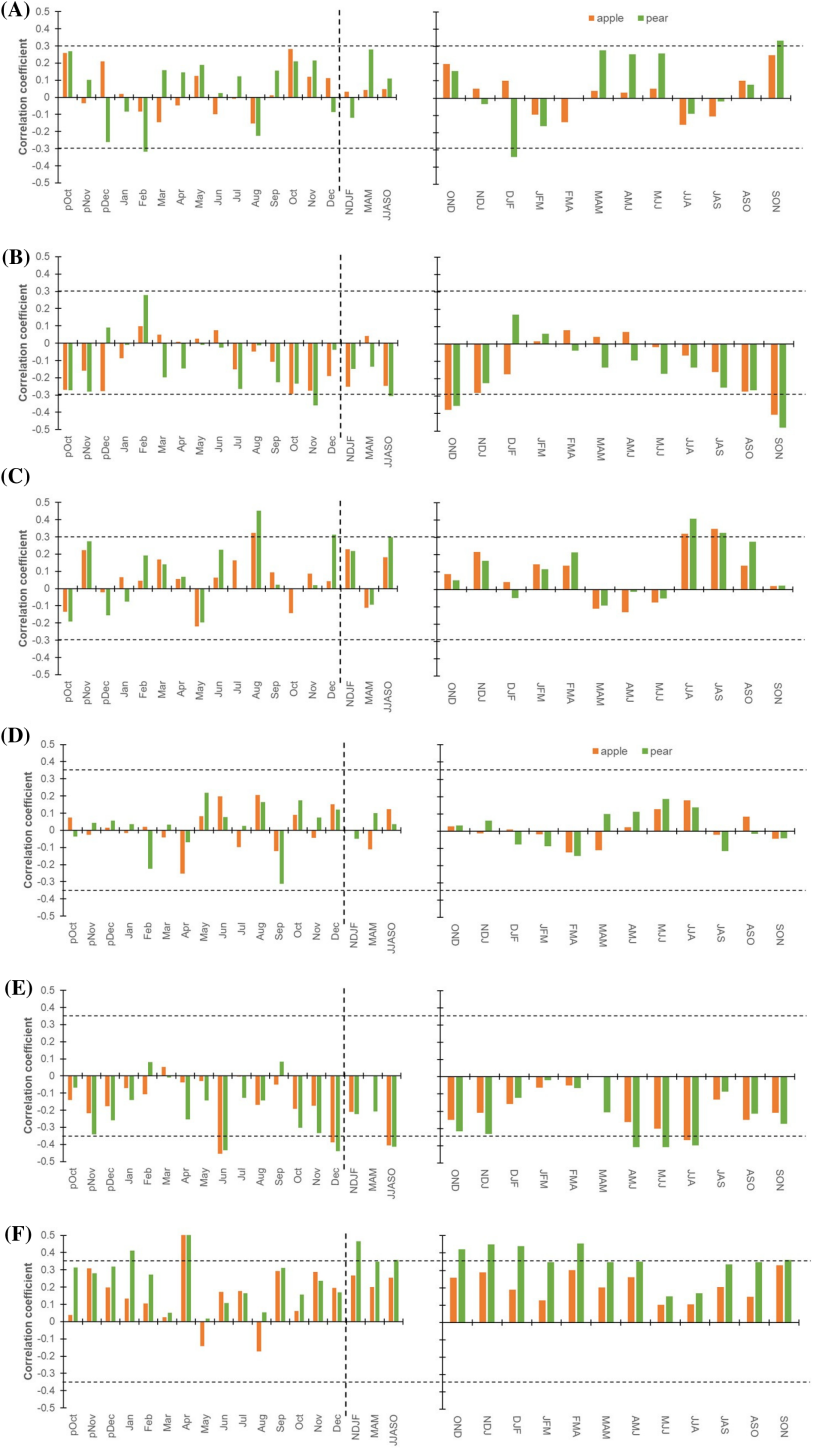
Hydro-climatic response function for Apple (orange) and Pear (green). **(A)** Monthly (left panel) correlations between δ^13^C time series of species and precipitation (1981-2021). The corresponding right panel indicates three-month moving correlation coefficients between δ^13^C and precipitation. **(B)** Monthly (left panel) and three-month moving (right panel) correlations between Δ^13^C and precipitation. **(C)** Monthly (left panel) and three-month moving (right panel) correlations between species’ C*i* chronologies and precipitation. **(D)** Monthly (left panel) correlations between δ^13^C chronologies of species and mean temperature (1988-2021). The corresponding right panel indicates three-month moving correlation coefficients between δ^13^C and mean temperature. **(E)** Monthly (left panel) and three-month moving (right panel) correlations between species Δ^13^C values and mean temperature. **(F)** Monthly (left panel) and three-month moving (right panel) correlations between species C*i* chronologies and mean temperature. The dotted horizontal line indicates a 95% confidence level. The dashed vertical line delimits months with seasonal aggregates. Prefix “p” before the months denotes the months of the previous growth year. The hydro-climatic response of species’ δ^13^C, Δ^13^C and C*i* to minimum and maximum temperature has been illustrated in **Supplementary Figure S2**.

*Hydro-climate response function*: the Pearson correlation with a response function approach demonstrates the relationship between species’ physiological processes (δ^13^C, Δ^13^C, and C*i*) and monthly hydroclimatic data (precipitation and temperature: mean, maximum, and minimum). To corroborate and stabilize these monthly relations, we plotted 3-month moving correlation coefficients (precipitation: 1982-2021; temperature: 1988-2021) (**Figure 4**). The response function analysis for the δ^13^C – precipitation relationship from October of the previous growth year to September of the current year (pOct-Sep) revealed non-significant correlations. The relationship confirms that water is not a limiting factor in these orchards, having provision of irrigation, especially for the apple trees. For this reason, at the beginning of the growing season (March-May) we observed an enhanced positive correlation for the pear trees. In contrast and irrespective of the seasons, δ^13^C – precipitation relationship for the apple trees was non-significant (**Figure 4A**). Likewise, Δ^13^C – and C*i* – precipitation relationship during peak growing season (June-October) was significant for the pear trees but non-significant for the apple trees having inverse correlations (**Figures 4B, C**). Yet, Pearson correlations and 3-month moving correlations with temperature indicated that the latter has a major control on the species’ ecophysiological processes (**Figures 4D–F**). The δ^13^C – temperature (mean) relationship was non-significant for both species (**Figure 4D**). Nevertheless, we observed significant, Δ^13^C – and C*i* – mean temperature correlations across the months for both species (**Figures 4E, F**). Particularly, the influence of minimum temperature on Δ^13^C and C*i* was prominent during the entire growing season (March – October) (**Supplementary Figures S2A–C**). Whereas, maximum temperature appears to affect these processes during the start of the growing season (April-June) (**Supplementary Figures S2D–F**). The relationships between temperature (Min., Max.) and δ^13^C, Δ^13^C, and C*i* have been detailed in the supplementary figure (**Supplementary Figures S2A–F**).

### 3.2 Temporal trends in oxygen isotope series and climate response

**Figure 5:**
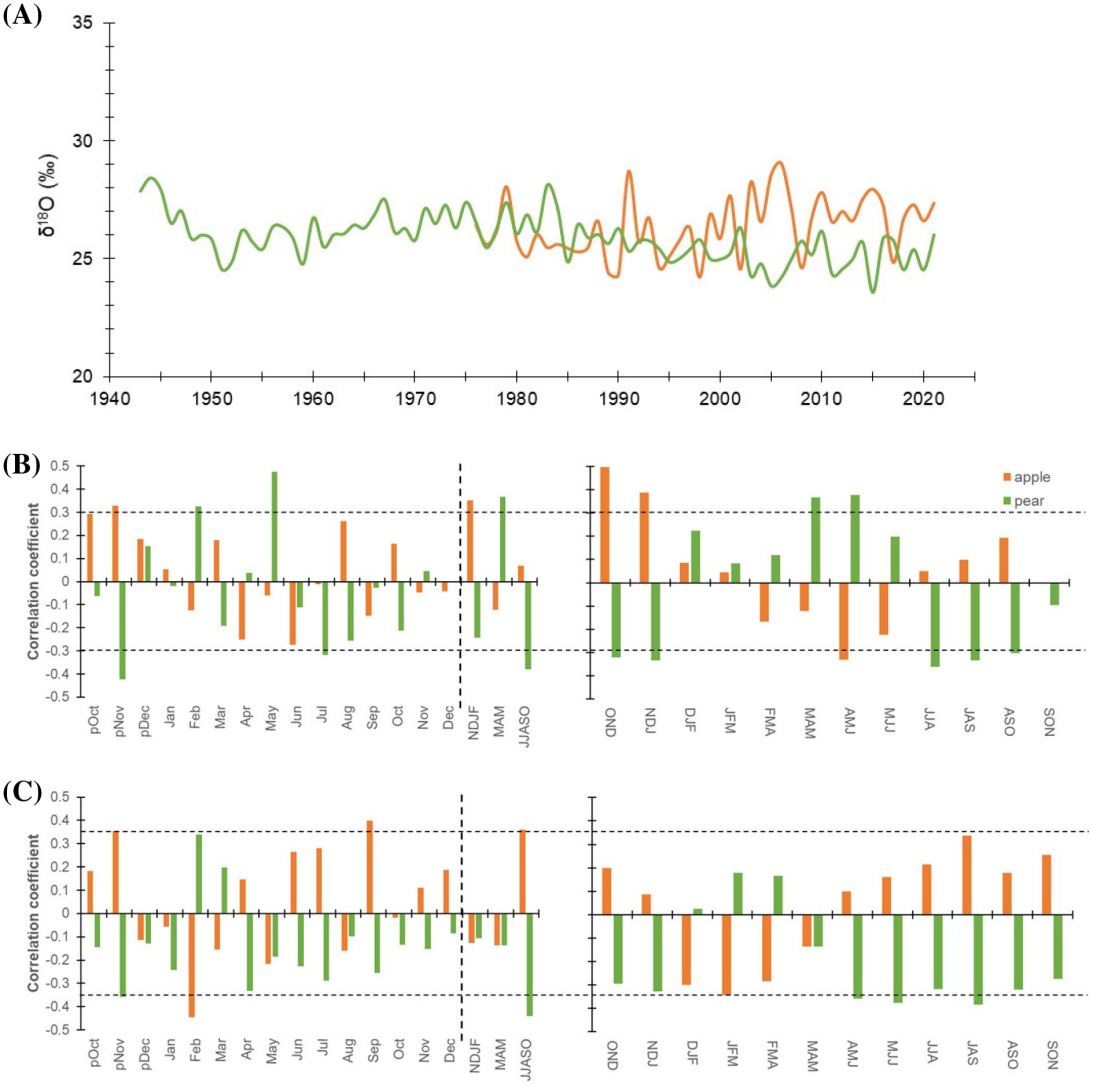
**(A)** δ^18^O isotope chronologies of apple (orange line) and pear (green line) tree species. **(B)** Monthly (left panel) correlations between δ^18^O chronologies of species and precipitation (1981-2021). The corresponding right panel indicates three-month moving correlation coefficients between δ^18^O and precipitation. **(C)** Monthly (left panel) and three-month moving (right panel) correlations between species’ δ^18^O chronologies and mean temperature (1988-2021). The dotted horizontal line indicates a 95% confidence level. The dashed vertical line delimits months with seasonal aggregates. Prefix “p” before the months denotes the months of the previous growth year. The response of species’ δ^18^O to minimum and maximum temperature has been illustrated in **Supplementary Figure S3**.

Oxygen isotope in the tree-rings complemented with respective δ^13^C values, remains the only proxy with proven potential to decipher a comprehensive picture of past and current ecophysiological status (Battipaglia et al., 2013; Nock et al., 2011; Siegwolf et al., 2023). Consequently, we have taken into account the δ^18^O chronologies of the trees (**Figure 5**). The mean δ^18^O value of pear trees (1943 – 2021) was 25.9 ± 0.99 ‰ (Coefficient of Variation (CV): 3.8%), while for the apple trees (1976 – 2021), it was ∼1.0 ‰ higher (26.3 ± 1.25 ‰) with a higher CV (5.6%) (**Table 1**). Prior to 1990, the mean δ^18^O of apple trees was 25.8 ‰ (range: 24.3 – 28.0 ‰), which showed a rising trend after 1990 with a similar range of variation. While, for the pear trees, the mean δ^18^O before 1990 was 26.3 ‰ (24.5 – 28.4 ‰), which dropped to 25.2 ‰ (23.5 – 26.3 ‰) since then (**Figure 5A**). The mean difference between species indicates a higher level of oxygen isotope discrimination and evapotranspiration in pear trees relative to the apple trees. At the same vapor pressure deficit, leaves with high transpiration rates are known to become isotopically enriched in heavy isotopes as compared to leaves having low transpiration (McCarroll and Loader, 2004; Battipaglia et al., 2013). The optimal transpiration rate coupled with the use of enriched surface irrigated water by the apple trees and differences in the stomatal conductance could be responsible for such an ^18^O enrichment offset. Moreover, because of limited evaporation, groundwater is less enriched (compared to surface water) and the probable use of this less enriched groundwater by the pear trees (having greater tree height and rooting depth) is reflected in its stable time series having lower CV (**Figure 5A**) and δ^13^C – precipitation relationship (**Figure 4A**). However, a decline (after 1990) in ^18^O enrichment (~1.0 ‰) in pear trees could be linked to increasing precipitation trend and irrigation provisioning.

**Table 1:** Descriptive statistics of tree-ring variables of the two tree species.

Species	Variables	Minimum	Maximum	Mean ± SE	SD
Apple (1976-2021)	δ^13^C (‰)	-26.6	-24.9	-25.6 ± 0.08	0.4
	Δ^13^C (‰)	17.1	19.4	18.0 ± 0.07	0.56
	C*i* (µmol mol^-1^ )	202.0	247.0	223.0 ± 1.96	10.3
	*i*WUE (µmol mol^-1^)	72.0	108.2	89.5 ± 1.23	8.8
	C*i*/C*a*	0.56	0.66	0.60 ± 0.003	0.02
	δ^18^O (‰)	24.1	29.0	26.3 ± 0.2	1.25
Pear (1943-2021)	δ^13^C (‰)	-27.7	-26.0	-26.6 ± 0.03	0.32
	Δ^13^C (‰)	18.6	20.6	19.6 ± 0.06	0.53
	C*i* (µmol mol^-1^ )	210.5	264.0	233.0 ± 1.47	13.6
	*i*WUE (µmol mol^-1^ )	55.3	91.5	70.4 ± 1.19	10.5
	C*i*/C*a*	0.63	0.72	0.67 ± 0.002	0.02
	δ^18^O (‰)	23.5	28.4	25.9 ± 0.11	0.99

Due to the above reasons, response function analysis for the monthly δ^18^O – precipitation relationship (pOct-Sep) showed opposite correlations in the species (**Figures 5B, C**). Cross-correlations of δ^18^O chronology of pear trees with precipitation revealed a strong positive relationship during the start of the growing season (March-May), which becomes negative during peak growing season (June-October). The δ^18^O – precipitation relationship for the apple trees, on the contrary, was opposite to that of the pear trees and insignificant throughout the growing season (March-October) (**Figure 5B**). Similarly, correlations with mean temperature indicated opposite but a major control on species’ ecophysiological processes associated with ^18^O enrichment, particularly during peak growing season (June-October). For this period, the correlation was significantly positive and negative for apple and pear trees, respectively (**Figure 5C**). In contrast, during March-May, correlation was negative in both species but remained statistically insignificant. Both Pearson and 3-month moving correlations between minimum temperature and δ^18^O have been detailed in the supplementary figure (**Supplementary Figures S3A, B**), which reflects greater but opposite response for the two species during peak growing season (June-October). On the contrary, the impact of maximum temperature was minimal. Modelling studies indicate that tree-ring δ^18^O values integrate signals from three primary factors: source water δ^18^O, evaporative enrichment of ^18^O in leaf water, and biochemical fractionation during organic matter synthesis. Consequently, tree-ring δ^18^O signals vary as a function of temperature, relative humidity, precipitation, water sources, and regional climate conditions (Barbour, 2007; Gessler et al., 2014; Kahmen et al., 2011). Based on the premise that tree roots take up soil water without fractionation, a major part of the isotopic signature in tree rings should reflect the variation in precipitation or hydroclimatic conditions (Farquhar et al., 2007; Lehmann et al., 2018; Roden et al., 2005). However, depending upon the local humidity condition, species-specific ecophysiological processes and responses (e.g.: isotopic composition of soil water, leaf-water enrichment, and oxygen isotope exchange reactions of photosynthates with water) may have a considerable effect on tree ring δ^18^O values (Baker et al., 2016; Lehmann et al., 2018), as revealed in our study.

### 3.3 Temporal trends in water use efficiency and climate controls

**Figure 6:**
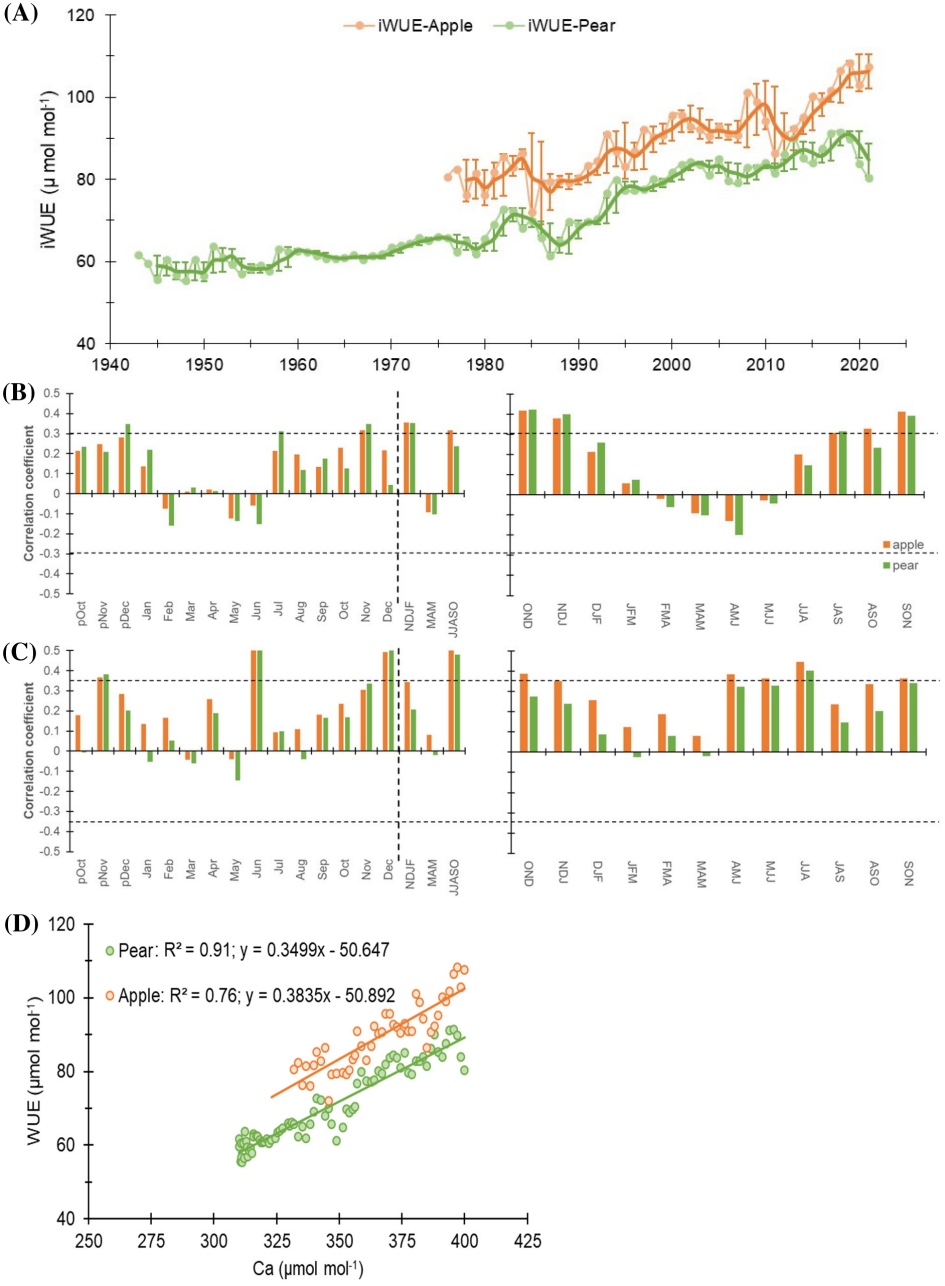
**(A)** *i*WUE time series of Apple and Pear. Light-colored lines with dots indicate annual values, while dark lines indicate 3-year moving average with std. dev. **(B)** Monthly (left panel) correlations between *i*WUE time series of species and precipitation (1981-2021). The corresponding right panel indicates three-month moving correlation coefficients between them. **(C)** Monthly (left panel) and three-month moving (right panel) correlations between species’ *i*WUE time series and mean temperature (1988-2021). The dotted horizontal line indicates a 95% confidence level. The dashed vertical line delimits months with seasonal aggregates. Prefix “p” before the months denotes the months of the previous growth year. The response of species’ *i*WUE to minimum and maximum temperature has been illustrated in **Supplementary Figure S4**. **(D)** Linear regression between *i*WUE and atmospheric CO_2_ concentration (C*a*) for apple and pear trees. Apple is slightly more sensitive to changes in C*a* (steeper slope), but Pear’s *i*WUE is more tightly correlated with atmospheric CO_2_ levels (higher R^2^).

Broadly, an increasing trend in *i*WUE was observed during the study period in both species that raised sharply after the 1990s (**Figure 6**). The mean *i*WUE of the apple tree was 88.5 ± 9.0 µmol mol^-1^, while for the pear trees, it was lower at 70.3 ± 10.5 µmol mol^-1^. Prior to 1990, the mean *i*WUE of apple trees was 80.2 µmol mol^-1^ (72 – 86 µmol mol^-1^), which increased to 93.6 µmol mol^-1^ (80 – 108 µmol mol^-1^). In contrast, for the pear trees, the mean *i*WUE before and after 1990 was 62.6 µmol mol^-1^ (55 – 73 µmol mol^-1^) and 81.8 µmol mol^-1^ (69 – 91 µmol mol^-1^), respectively. During the observation period, mean *i*WUE of the apple trees was higher than that of the pear trees (~18 µmol mol^-1^), which after 1990 increased by 15.5 and 30.6% respectively for apple and pear trees (**Table 1**). For the common period, inter-species correlation was significantly high (*r* = 0.82, *p*< 0.001), which probably indicates the predominant control of climate on species’ ecophysiological process.

Moreover, it would be informative to note that long-term gains in *i*WUE by vegetation are usually overestimated (Gong et al., 2022). To provide a more accurate assessment, it is crucial to account for post-photosynthetic fractionations and mesophyll conductance, which influence CO_2_ diffusion to carboxylation sites (Gimeno et al., 2021; Gong et al., 2022; Ma et al., 2021). Our *i*WUE calculations, based on a linear model of photosynthetic ¹³C discrimination (Δ¹³C), do not fully capture long-term structural and physiological acclimations. Therefore, we advocate for the adoption of advanced models that should incorporate post-photosynthetic fractionations and mesophyll conductance as well as photorespiration to mitigate errors in estimating *i*WUE from Δ¹³C across vegetation types.

Nevertheless, to elaborate on the climatic controls of *i*WUE, we performed response function analysis on *i*WUE chronologies and monthly hydroclimatic datasets. For both species, major hydroclimatic variables (temperature and precipitation) showed positive relations with *i*WUE (**Figures 6B, C**). During the beginning of the growing season (March-May), *i*WUE – precipitation relationship was negative (*p* > 0.05) for the species that turned to positive correlations during the peak growing season (June-October) (**Figure 6B**). Cross-correlations with mean temperature revealed a strong positive correlation during peak growing season in both species, which was insignificantly low during March to May (**Figure 6C**). Similarly, with respect to minimum and maximum temperature, we noted a higher influence of minimum temperature on *i*WUE during peak growing season in both species (**Supplementary Figures S4A, B**).

Furthermore, stepwise regression between annual and 5-year mean *i*WUE (as dependent variable) and mean temperature (Tmean), precipitation (Ppt), and atmospheric CO_2_ concentrations (CO_2_) (as independent variables) were employed to access the explanatory power of climate variables. In both species, annual CO_2_ explained more than 70% of the variability. Whereas, 5-year mean CO_2_ explained more than 90% of the variability in *i*WUE. Analyses further reveal that Tmean is the second most important variable, while precipitation has a minimal impact on species’ *i*WUE (**Supplementary Table S1**). Several studies have inferred that rising atmospheric CO_2_ levels do not always imply enhanced photosynthetic rate and tree growth (Nock et al., 2011; Rahman et al., 2020; Van Der Sleen et al., 2015), with species-specific responses. We also observed species-specific responses and noted a differential response of species’ *i*WUE to atmospheric CO_2_ (C*a*) (**Figure 6D**). The regression coefficient in apple trees (R^2^ = 0.76) was lower than that of the pear trees (R^2^ = 0.91). Results further suggest a slightly higher sensitivity of apple trees to rising C*a* (slope: 0.38). However, a lower regression coefficient in apple trees indicates the possible role of other environmental factors particularly soil moisture (via stomatal regulation) in influencing their *i*WUE response. On the other hand, pear trees’ *i*WUE appears more tightly correlated with atmospheric CO_2_ levels (higher R^2^) possibly due to their access to relatively invariable source water (groundwater), but having a slightly lower slope (0.35). Despite this, both species have similar intercepts (Apple: -50.89 and Pear: -50.64), suggesting comparable baseline *i*WUE in the context of CO_2_ response. This implies that both species started from a similar baseline, but their responses to rising CO_2_ levels have diverged.

In conclusion, both species have responded to rising CO_2_ levels, with apple trees showing higher sensitivity as well as variability to environmental changes. Pear trees, on the other hand, have exhibited a more predictable and consistent response. It’s possible that apple trees may have reached a threshold in their ability to increase WUE as CO_2_ rises (**Figure 6D**). This information is valuable for predicting how these species may adapt to future environmental conditions, especially in regions with fluctuating water availability.

### 3.4 Temporal trends in species’ dual isotope (δ^13^C – δ^18^O) series

**Figure 7:**
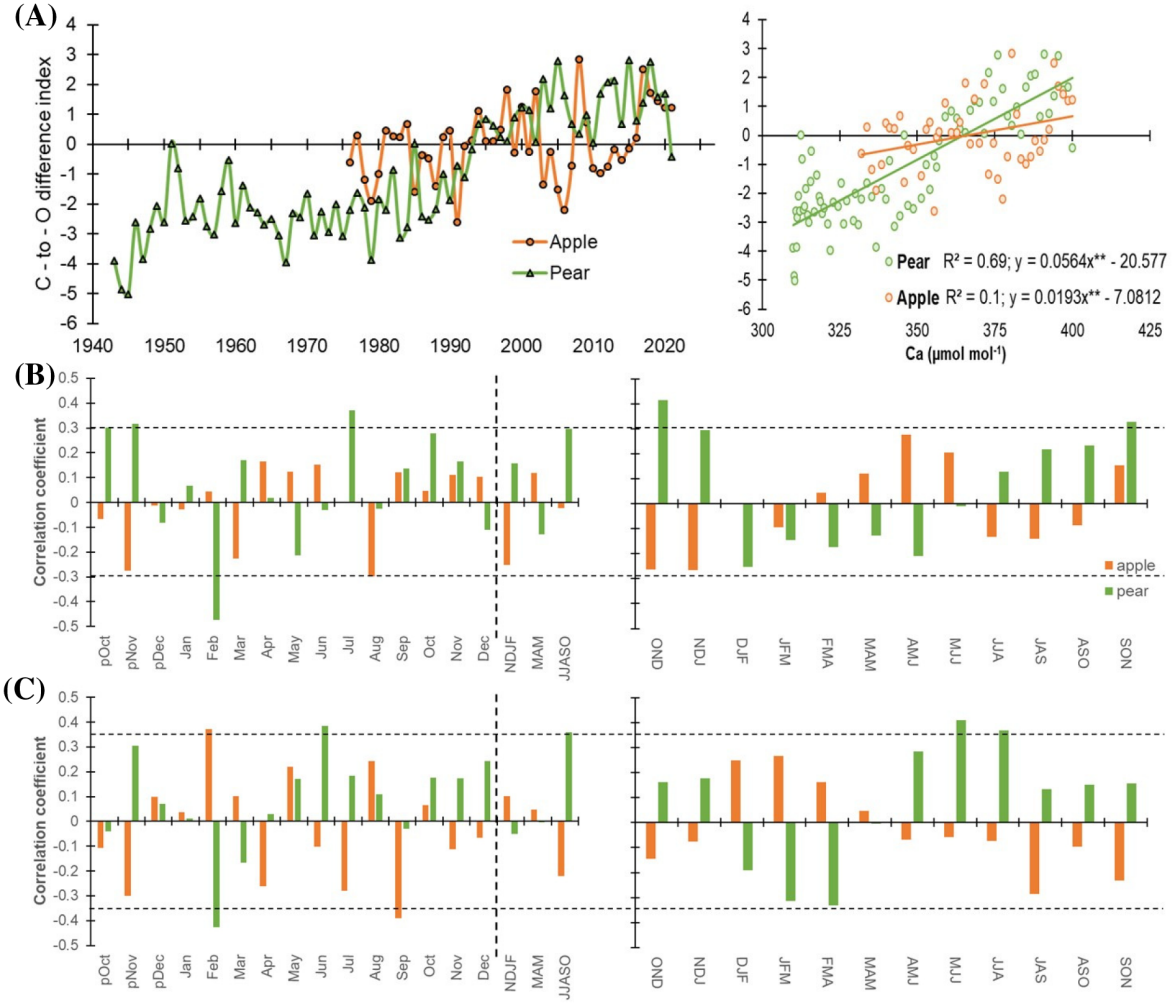
**(A)** Standardized carbon-to-oxygen isotope difference index for apple (orange) and pear (green) for the corresponding observation periods. Right panel plots the C-to-O difference index with atmospheric CO_2_ levels (C*a*) indicating physiological responses (through changes in stomatal conductance and photosynthetic capacity) to rising C*a*. Slopes of both species are significant (**p< 0.0001). **(B)** Monthly (left panel) correlations between index time series of species and precipitation (1981-2021). The corresponding right panel indicates three-month moving correlation coefficients between them. **(C)** Monthly (left panel) and three-month moving (right panel) correlations between species’ index time series and mean temperature (1988-2021). The dotted horizontal line indicates a 95% confidence level. The dashed vertical line delimits months with seasonal aggregates. Prefix “p” before the months denotes the months of the previous growth year. The response of species’ index time series to minimum and maximum temperature has been illustrated in **Supplementary Figure S5**.

Tree-ring δ^18^O, in combination with δ^13^C, is a powerful proxy to decipher a comprehensive picture of past and current ecophysiological status (Battipaglia et al., 2013; Nock et al., 2011; Siegwolf et al., 2023). Therefore, we computed carbon-to-oxygen isotope difference index for both species (**Figure 7A**). This difference serves as an indicator of the trees’ physiological conditions related to changes in stomatal conductance and photosynthetic capacity (Scheidegger et al., 2000).

The C-to-O difference index for apple trees exhibits an increasing trend from the late 1970s onwards, with a more pronounced positive shift after the year 2000. The index also shows considerable year-to-year variability. In the early years (1976-1980), values fluctuate around the zero line, indicating a balance between low stomatal conductance and high photosynthetic capacity. From 1980 onwards, periods of high photosynthetic capacity (positive index values) appear more frequent and sustained, likely reflecting the irrigation effect. In recent years (2000-2021), the index mostly remained positive, indicating a generally high photosynthetic capacity for apple trees, despite occasional dips around 2010 and 2015.

The index for pear trees shows an overall increasing trend over the entire period. There is a clear positive shift around the late 1980s, aligning with the apple tree index. However, the species’ pattern briefly diverged during the 2010s when δ^13^C levels were stable and δ^18^O values were high in apple trees (**Figures 2**, **5**). Similar to apple trees, pear trees exhibit considerable inter-annual variability. The period before 1980 shows more frequent negative values, indicating periods of low photosynthesis, likely induced by stomatal limitations given limited irrigation provisioning. Post-1980, positive values become more dominant, suggesting an improvement in photosynthetic assimilation. In recent decades (2000-2021), the overall trend remained positive, with a few dips indicating brief periods of moisture stress and reduced photosynthetic efficiency. The analysis suggests an overall improvement in photosynthetic capacity over the studied periods, with more frequent and sustained positive index values in recent decades in both species. This trend reflects adaptive physiological responses to changing environmental conditions including the CO_2_ fertilization effect in the Italian Alps. However, both species exhibit notable year-to-year variability, highlighting the influence of inter-annual climatic variations on tree physiology and productivity.

Previously, we observed species-specific responses of *i*WUE to atmospheric CO_2_ (C*a*), with pear trees exhibiting lower *i*WUE and a higher regression coefficient (R² = 0.91) compared to apple trees (R² = 0.76) (**Figures 6A, D**). To further explore these differences, we plotted species’ C-to-O difference indices against C*a* (**Figure 7A**). The slope of pear trees (0.056, *p*< 0.001) with respect to apple trees (0.019, *p*< 0.001) suggests a stronger physiological response to increased CO_2_, with clear changes in stomatal conductance and photosynthetic activity (**Figure 7A**). Further, a much higher R² value for pear trees (69%) indicates that a significant portion of the changes in stomatal conductance and photosynthetic activity can be explained by changes in CO_2_ concentration.

This suggests that pear trees’ physiological conditions, as indicated by the index, are more closely tied to rising CO_2_ levels compared to apple trees. The weak slope and low regression coefficient suggest that the physiological processes in the apple tree are primarily related to the stomatal regulation via soil moisture. Overall, our analysis revealed a significantly higher correlation for pear trees (r = 0.83, *p*< 0.0001) than for apple trees (r = 0.32, *p*< 0.05). These results suggest that apple trees may have reached a threshold in their ability to increase water use efficiency as CO_2_ levels rise, while pear trees continue to show a stronger response. This could explain the differential responses of these two tree species to changing environmental conditions, including the CO_2_ fertilization effect.

Cross-correlations with hydroclimatic variables were further performed to gain insights into the combined responses for the index series of the species. Response analyses clearly indicate that precipitation has a minimal impact on isotope-inferred ecophysiological processes, except for the pear trees which show a significant positive correlation during peak growing season (**Figure 7B**). Similarly, index – mean temperature relationship was significant only for the pear trees that only during peak growing season (**Figure 7C**). Both Pearson and 3-month moving correlations between minimum/maximum temperature and species index series have been detailed in the supplementary figure (**Supplementary Figures S5A, B**). These results demonstrate hydroclimate and ecophysiological relationships in these energy-limited ecosystems.

## 4 Conclusion

This study utilized unique old growth veteran apple and pear trees from energy-limited alpine valleys of the Italian Alps (a major national production center) to investigate climate-scale physiological responses. Results broadly indicate a species-coherent behavior and trends in δ^18^O and processes such as *i*WUE, carbon discrimination (*Δ* ^13^C) and intercellular CO_2_ concentration (*C*i). Importantly, results suggest a similar physiological response of tree species to atmospheric CO_2_ rise. A significant increase in *i*WUE has been observed in recent decades, primarily driven by the CO_2_-fertilization effect. Dual isotope analyses (δ^18^O–δ^13^C) confirmed that the recent rise in *i*WUE is due to increased carbon assimilation rather than reduced evapotranspiration. The analyses also highlight common inter-annual variability in carbon assimilation across both species, with some site- and species-specific responses. Among the major climatic controls on ecophysiological processes, precipitation has minimal impact in this moist, energy-limited ecosystem. Statistical and climate response function analyses further revealed that, besides CO_2_-fertilization, the second most important environmental driver of ecophysiological processes is the minimum temperature during the growing season. We believe that such long-term records could be valuable for fine-tuning land surface models to account for the combined effects of CO_2_-fertilization and climate impact.

## Data Availability

The raw data supporting the conclusions of this article will be made available by the authors, without undue reservation.
