# Supplementary material for: Multi-decadal tree-ring stable isotope records of apple and pear trees indicate coherent ecophysiological responses to environmental changes in alpine valleys
Source: Front Plant Sci. 2025 Jan 10;15:1471415. doi: 10.3389/fpls.2024.1471415 (PMC11757252; doi:10.3389/fpls.2024.1471415)
Supplement: Supplementary file 1 [file SupplementaryFile1.zip › Zipped_Supplementary/Supplementary Figure.docx]

**Supplementary Figures**

| 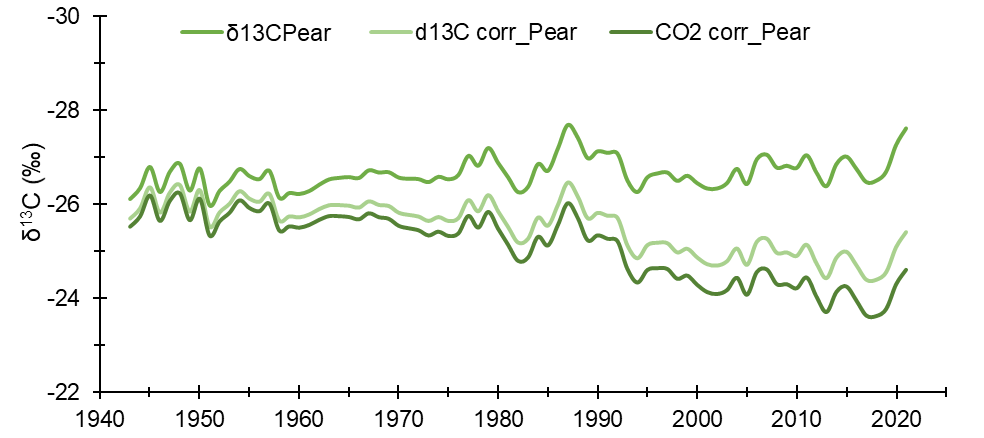  **(A)** |
| --- |
| 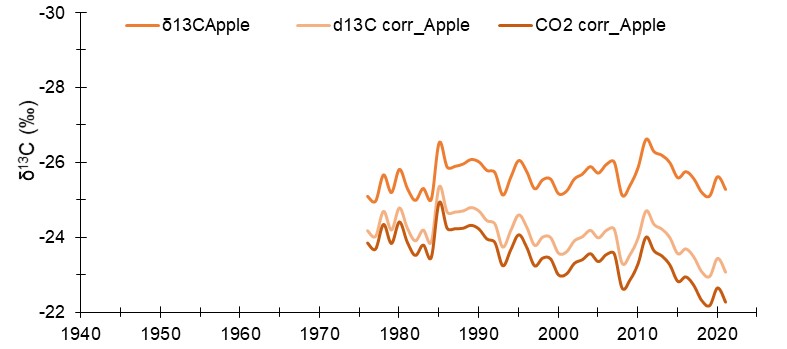  **(B)** |
| Fig. S1. Comparison of raw δ^13^C series (bold lines), δ^13^C series corrected for post-industrial changes in isotopic composition of atmospheric CO_2_ (bright lines), and δ^13^C series after additional correction for physiological responses to increasing concentrations of atmospheric CO_2_ (dark lines) in pear (A), and apple trees (B). |

**(A)**

| 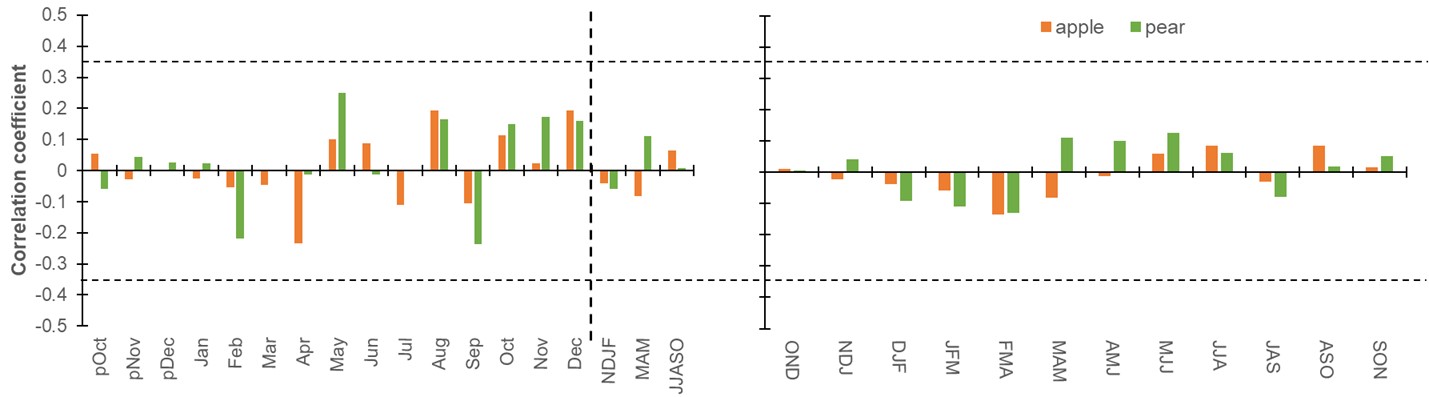 |
| --- |
| 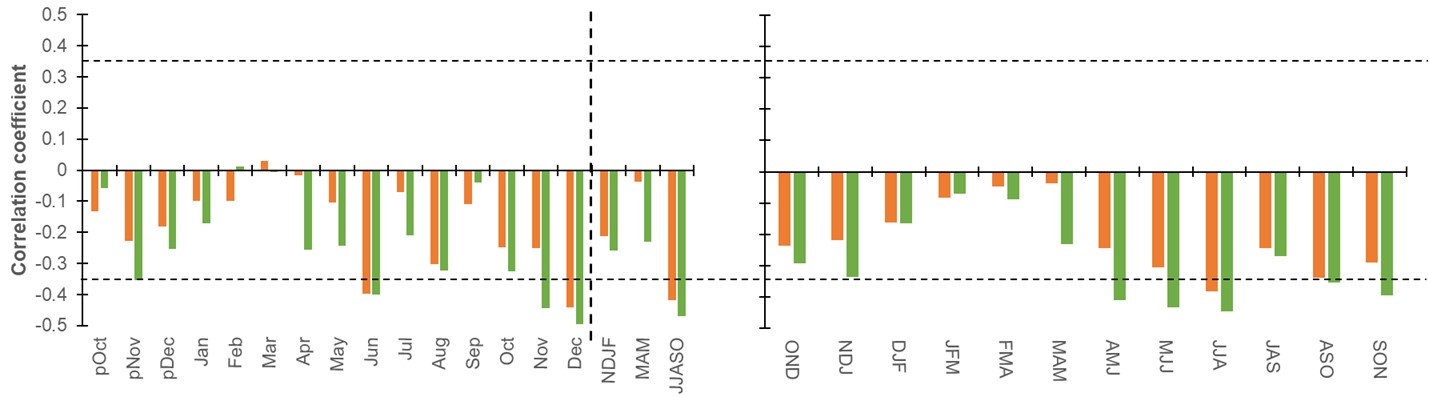  **(B)** |
| 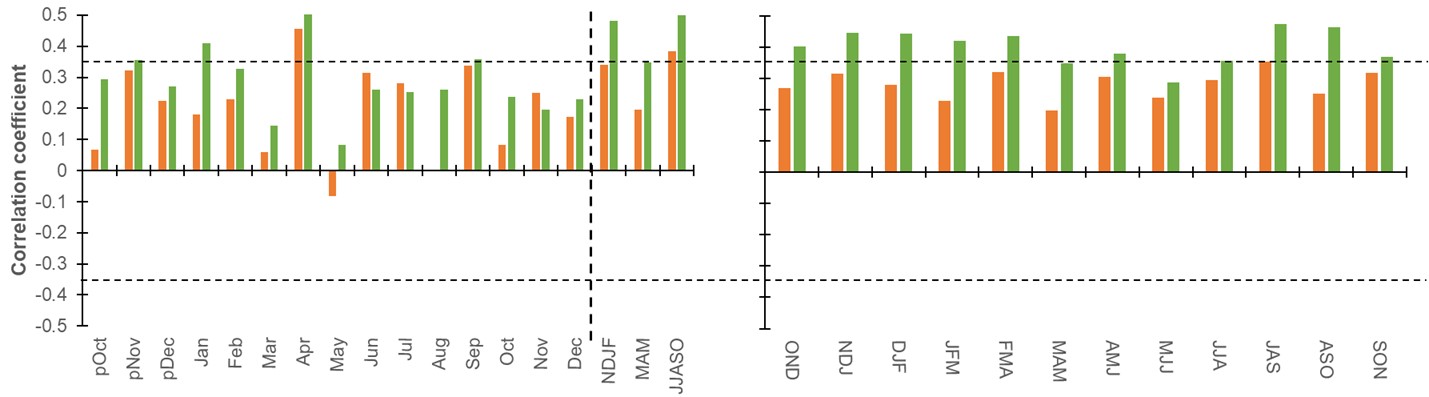  **(C)**  **(D)** |
| 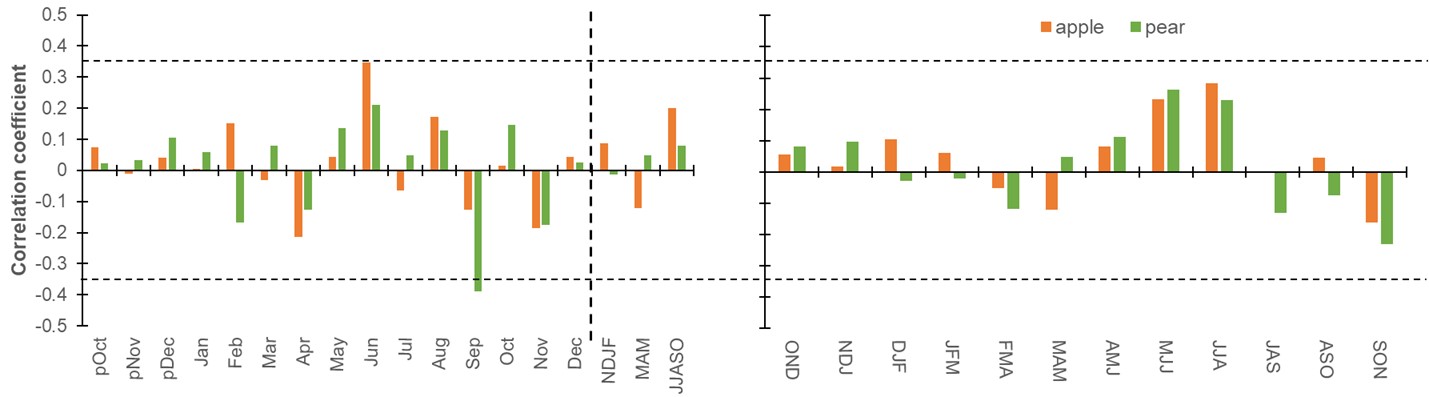 |
| 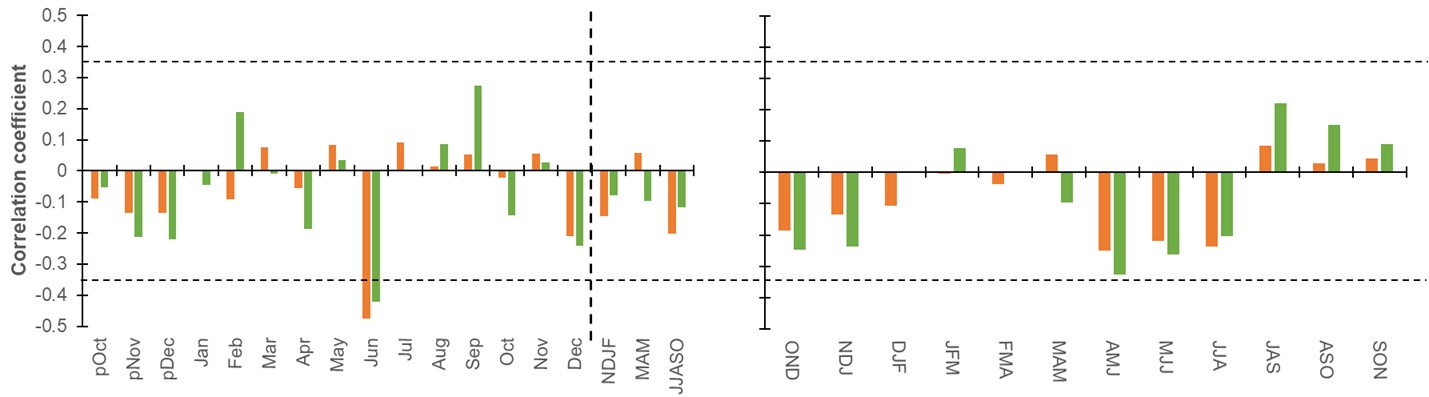  **(E)** |
| 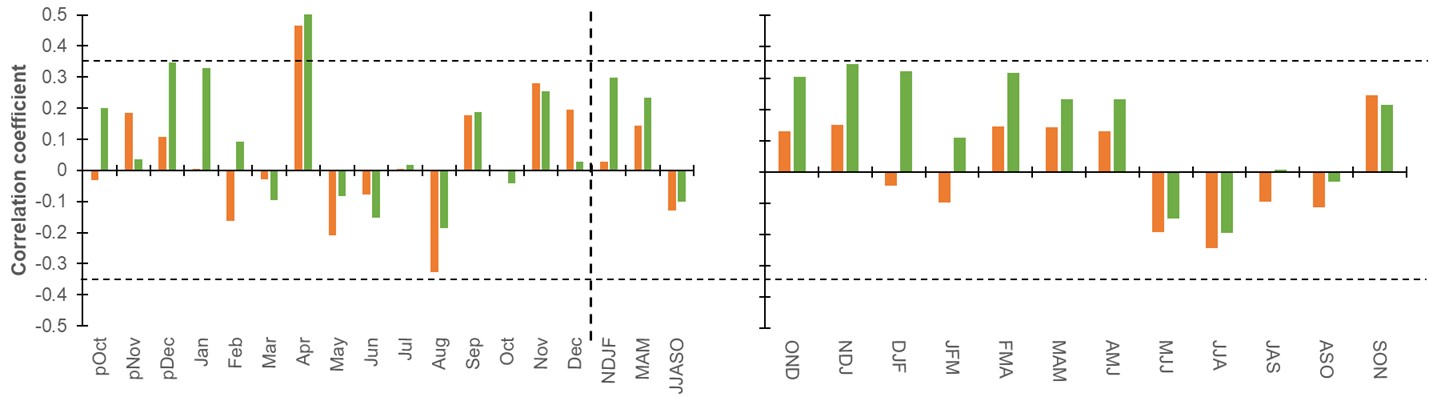  **(F)** |
| Fig. S2. The hydro-climatic response of species’ δ^13^C to minimum temperature (1988-2021) (A), Δ^13^C and minimum temperature (B), C*i* and minimum temperature (C). (D) δ^13^C and maximum temperature. (E) Δ^13^C and maximum temperature, (F) C*i* and maximum temperature (Apple: orange; Pear: green). The dotted horizontal line indicates a 95% confidence level. The dashed vertical line delimits months with seasonal aggregates. Prefix “p” before the months denotes the months of the previous growth year. |

**(A)**

| 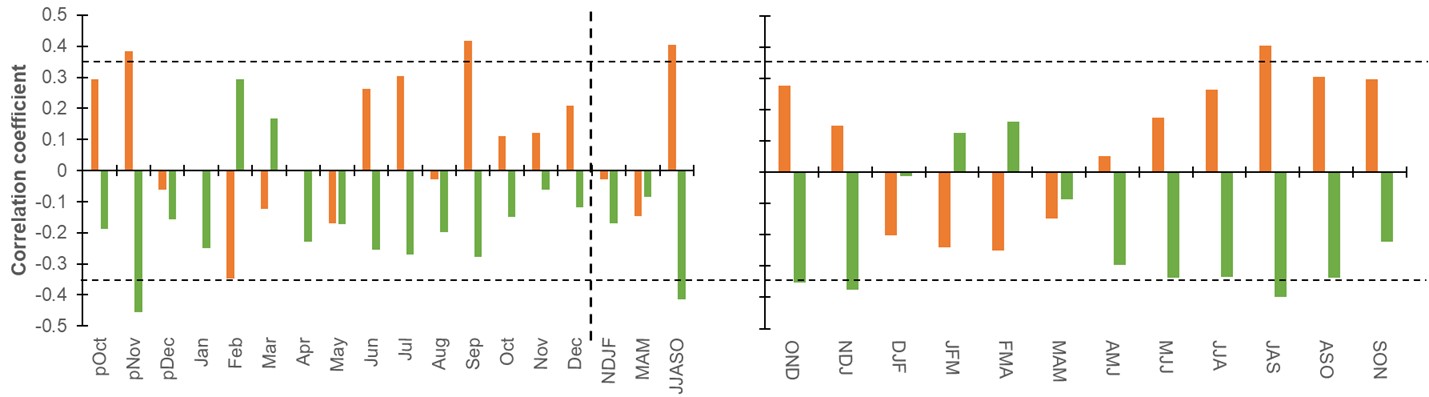 |
| --- |
| 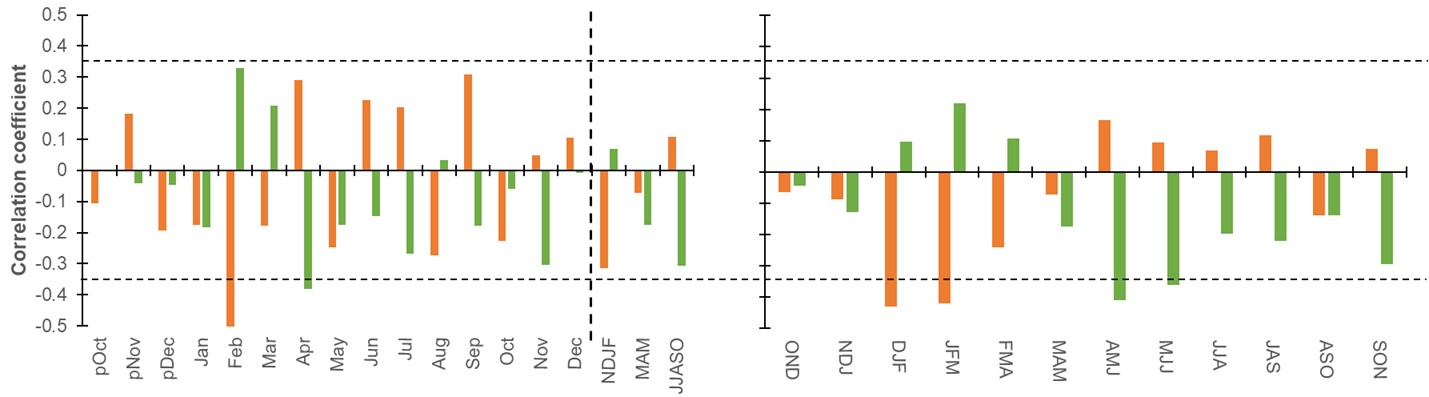  **(B)** |
| Fig. S3. The response of species’ δ^18^O to minimum (A) and maximum temperature (B) (Apple: orange; Pear: green). The dotted horizontal line indicates a 95% confidence level. The dashed vertical line delimits months with seasonal aggregates. Prefix “p” before the months denotes the months of the previous growth year. |

| 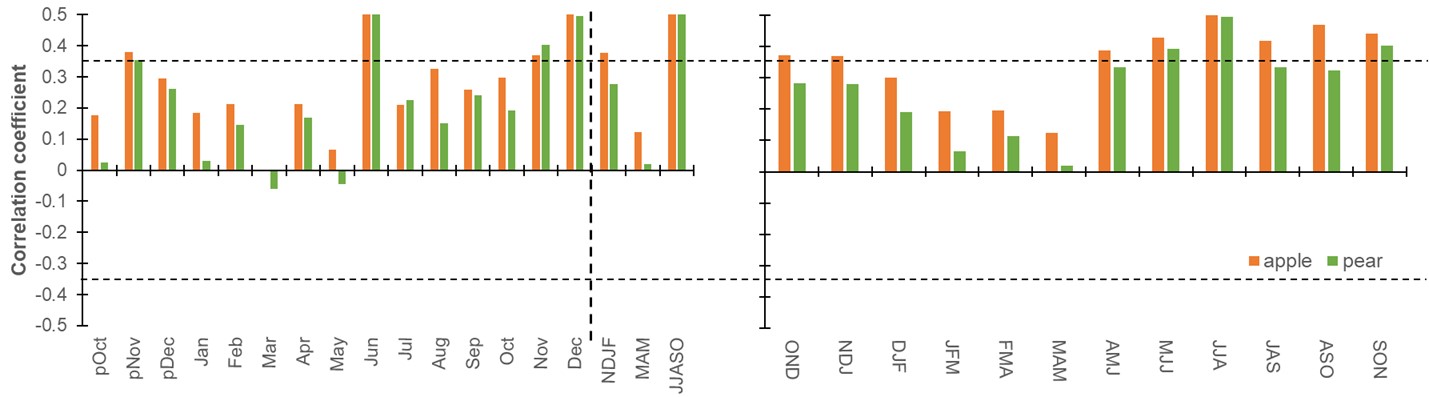  **(A)** |
| --- |
| 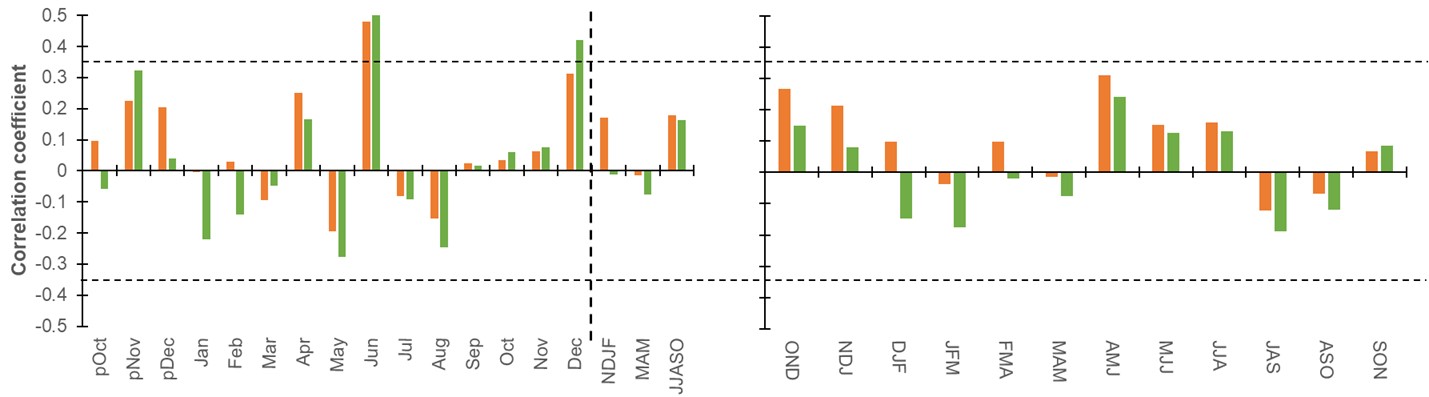  **(B)** |
| Fig. S4. The response of species’ *i*WUE time series to minimum (A) and maximum temperature (B) (Apple: orange; Pear: green). The dotted horizontal line indicates a 95% confidence level. The dashed vertical line delimits months with seasonal aggregates. Prefix “p” before the months denotes the months of the previous growth year. |

| 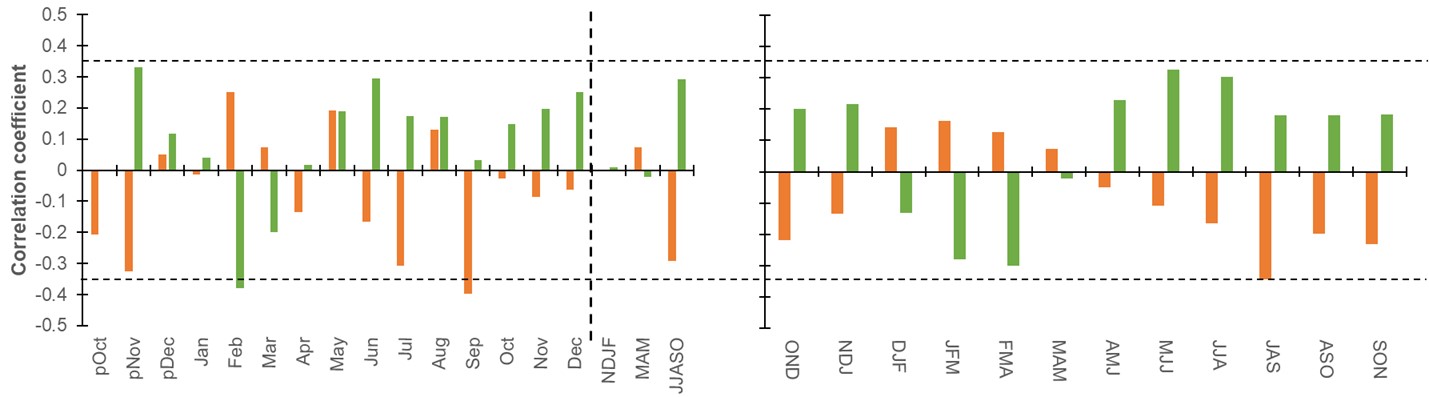  **(A)** |
| --- |
| 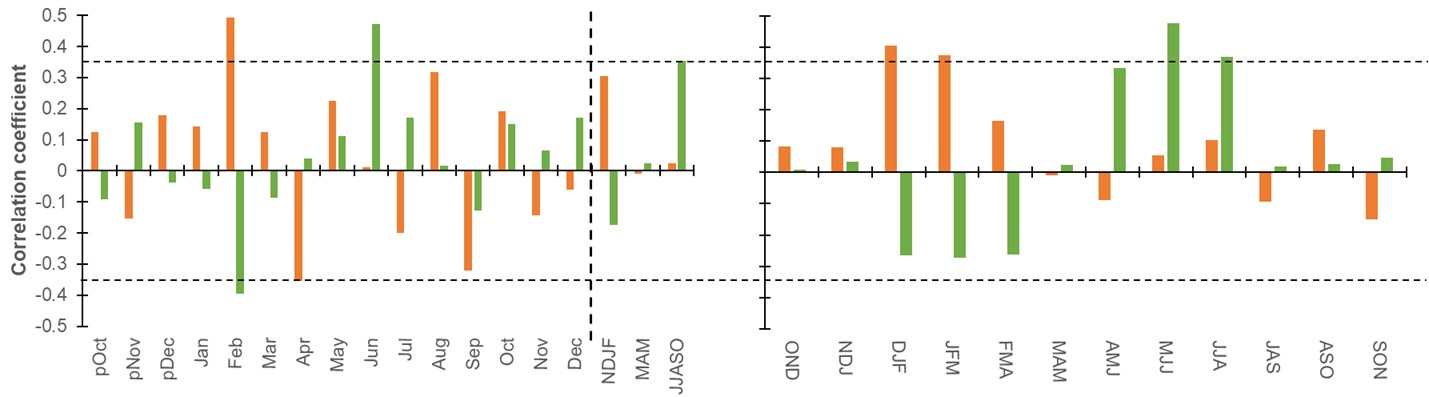  **(B)** |
| Fig. S5. The response of species’ carbon-to-oxygen isotope difference index to minimum (A) and maximum temperature (B) (Apple: orange; Pear: green). The dotted horizontal line indicates a 95% confidence level. The dashed vertical line delimits months with seasonal aggregates. Prefix “p” before the months denotes the months of the previous growth year. |
