# Supplementary material for: Multi-decadal tree-ring stable isotope records of apple and pear trees indicate coherent ecophysiological responses to environmental changes in alpine valleys
Source: Front Plant Sci. 2025 Jan 10;15:1471415. doi: 10.3389/fpls.2024.1471415 (PMC11757252; doi:10.3389/fpls.2024.1471415)
Supplement: Supplementary file 1 [file SupplementaryFile1.zip › Zipped_Supplementary/Supplementary Table.docx]

**Supplementary Table**

**Table S1a**. Stepwise regression estimates for the species: δ^13^C-derived *i*WUE is the dependent variable and annual mean temperature (Tmean), precipitation (Ppt), and atmospheric CO_2_ concentrations (CO_2_) are the independent variables.

| Species | Parameter | Estimate | *P* – value | R^2^ |
| --- | --- | --- | --- | --- |
| Apple | CO_2_ | 0.43 | 0.000 | 0.73 (*P* < 0.001) |
|  | Tmean | -0.51 | 0.618 |  |
|  | Ppt | 0.007 | 0.295 |  |
| Pear | CO_2_ | 0.40 | 0.000 | 0.77 (*P* < 0.001) |
|  | Tmean | -1.05 | 0.223 |  |
|  | Ppt | 0.006 | 0.273 |  |

**Table S1b**. Stepwise regression estimates for 5-year mean: 5-year mean *i*WUE is the dependent variable and 5-year mean temperature (Tmean), precipitation (Ppt), and atmospheric CO_2_ concentrations (CO_2_) are the independent variables.

| Species | Parameter | Estimate | *P* – value | R^2^ |
| --- | --- | --- | --- | --- |
| Apple | CO_2_ | 0.53 | 0.021 | 0.95 (*P* = 0.01) |
|  | Tmean | -5.54 | 0.225 |  |
|  | Ppt | 0.02 | 0.200 |  |
| Pear | CO_2_ | 0.50 | 0.072 | 0.91 (*P* = 0.04) |
|  | Tmean | -5.09 | 0.377 |  |
|  | Ppt | 0.013 | 0.504 |  |
